# A Long Slit-Like Entrance Promotes Ventilation in the Mud Nesting Social Wasp, *Polybia spinifex*: Visualization of Nest Microclimates using Computational Fluid Dynamics

**DOI:** 10.1673/031.010.14135

**Published:** 2010-10-11

**Authors:** Satoshi Hozumi, Terumi Inagaki

**Affiliations:** ^1^ Venture Business Laboratory, Graduate School of Science and Engineering, lbaraki University, 316-8511, Japan; ^2^ Graduate School of Science and Engineering, lbaraki University, 316-8511, Japan

**Keywords:** computational fluid dynamics, nest architecture

## Abstract

*Polybia spinifex* Richards (Hymenoptera: Vespidae) constructs mud nests characterized by a long slit-like entrance. The ventilation and thermal characteristics of the *P. spinifex* nest were investigated to determine whether the nest microclimate is automatically maintained due to the size of the entrance. In order to examine this hypothesis, a numerical simulation was employed to predict the effects of the entrance length. The calculations were performed with 3D-virtual models that simulated the *P. spinifex* nest conditions, and the reliability of the simulations was experimentally examined by using gypsum-model nests and a *P. spinifex* nest. The ventilation effect was determined by blowing air through the nest at 1–3 m/s (airflow conditions); the airspeed was found to be higher in models with a longer entrance. The ventilation rate was also higher in models with longer entrances, suggesting that the *P. spinifex* nest is automatically ventilated by natural winds. Next, the thermal effect was calculated under condition of direct sunlight. Under a calm condition (airflow, 0 m/s), thermal convection and a small temperature drop were observed in the case of models with a long entrance, whereas the ventilation and thermoregulation effects seemed small. Under airflow conditions, the temperature at the mid combs steeply dropped due to the convective airflow through the entrance at 1–2 m/s, and at 3 m/s, most of the heat was eliminated due to high thermal conductivity of the mud envelope, rather than convection.

## Introduction

Social wasps have developed a number of behavioral adaptations to deal with seasonal and daily variations in climate parameters. At the beginning of colony life, foundresses or workers in swarms choose a suitable location to nest (Morimoto 1953; Kudô and Zucchi 2009). When this location is identified, the workers build and modify the nest such that automated control of the microclimate is achieved to a certain extent. Such automated regulations are notable in the elaborate nests of termites (Heinrich 1993; Korb 2003) and ants (Banschbach et al. 1997; Kleineidam et al. 2001); both ventilation and temperature are regulated in their nests. In social wasp nests, temperature-regulative nest architectures have been reported; for example, envelopes, extra cells, or combs are built to increase nest insulation in vespine wasps (Spradbery 1973), *Polistes* (Hozumi and Yamane 2008) and *Polybia* species (Hozumi et al. 2008). However, to our best knowledge, no studies have revealed automatic ventilation in social wasp nests.

The genus *Polybia* belongs to a polistine tribe Epiponini, which is endemic in the Neotropical region (Carpenter 1991). In this genus, which includes 56 described species, populations in a colony vary from hundreds to tens of thousands of individuals. The wasps build characteristic nests in modules, with each module consisting of a comb of cells plus the envelope covering the comb. Each envelope serves as a substrate for the cells of the next comb. The nest architecture is called phragmocyttarous (Richards and Richards 1951; Jeanne 1975). The *Polybia* wasps commonly use wood pulp or plant fibers as the main raw material to construct nests (Richards and Richards 1951; Wenzel 1991); of these, three species in the subgenus Pedothoeca, and one species in the subgenus Furnariana, share the derived trait of mud nest construction (Richards 1978; Jeanne 1991; Cooper 1993). The use of mud for nest construction has an ecological advantage in that the hard mud nest is more resistant to destruction by vertebrate predators (O'Donnell and Jeanne 2002) and heavy rains (Richards 1978) than the paper nest. In the mud nests, the thermal conductivity (0.69 W/(m°C)) is much higher than those of paper nests (Hozumi et al. 2009); a paper envelope of a vespine nest is 0.08–0.20 W/(m°C) (Schmolz et al. 2000).

**Figure 1.**
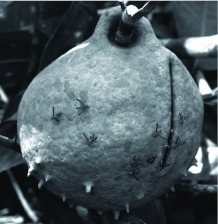
A natural nest of *Polybia spinifex*. High quality figures are available online.

*Polybia spinifex* Richards (Hymenoptera: Vespidae) belongs to subgenus Pedothoeca, whose members build mud nests with characteristic entrances (Richards 1978). The mud envelope has a narrow and vertically long slit down one side as an entrance, which provides access to the combs (Figure 1). The internal architecture of the nest is otherwise typical of the genus, i.e., phragmocyttarous. However, the combs are not completely enclosed and are slightly emarginated with a rounded tip opposite the entrance slit, forming an entrance hall built in wellhole style. Since *P. spinifex* often choose open and sunny sites, such as riverbanks (Richards 1978), for nesting, the nests receive the sunlight and rain directly. Other *Polybia* mud nesters build on high branches in the forest (2–20m above the ground; Richards 1978). Compared with other mud nesters belonging to Pedothoeca, such as *P. singularis* and *P. emaciata*, the architecture of the *P. spinifex* nest is unique in having a few spine-like projections, which may serve as conduits for raindrops (Figure 1).

There are 2 important ecological problems with the *P. spinifex* nests, namely, nest defense and climate regulation, i.e., nest ventilation and thermoregulation. In general, the nest entrance is the boundary between the nest and external environment, and the entrance size is such that only adult wasps can pass through it, thereby preventing the entry of predators and parasitoids (Spradbery 1973). In the *P. spinifex* nests, the entrance is very long (> 10 cm); it is 20 times longer than the height of the adults, and the width is slightly larger than that of the adults. Such a large hole may be less defensive against infestations by phorid flies (London and Jeanne 1998). From the viewpoint of microclimatic regulation, on the other hand, the nest is probably easily filled with CO_2_ due to the high respiratory metabolism of the colony (Hebling-Beraldo et al. 1990) because the mud envelope is highly airtight and does not allow vapor to pass through. In hot tropical climates, the nest is easily overheated due to insolation by receiving direct sunlight; since the thermal conductivity of mud envelope is very high, it conducts heat into the nest as soon as sunlight hits it (Hozumi et al. 2009). These architectural features of the *P. spinifex* nest seem to adversely affect colony life. It is considered that the long slit-like entrance plays a role in relation to microclimate regulation; however, the functions of the entrance have not yet been studied.

In principal, there are 2 main forces driving nest climate regulation: active regulation by adults and passive regulation via the nest architecture. Here, passive regulation includes ventilation by temperature-induced (thermalconvection) and wind-induced (forcedconvection) airflow, which are also strongly related to the thermal conditions of the nest. This study aimed to understand the functions of the entrance in passive-microclimate regulation in the nest. The methods of computational fluid dynamics (CFD) were employed to estimate and visualize the nest microclimates, and the relevance of the calculations was tested with experimental measurements.

## Materials and Methods

### Preparation of the virtual 3D-model nest and calculation domain

Computational fluid dynamics (CFD) is a numerical analysis and simulation procedure that enables the observation of flow via equations for calculating the movement of fluid, such as the conservation of mass equation, energy conservation equation, and Navier-Stokes equations. Simulations with CFD are advantageous as they facilitate nondestructive assessment of the nest climates and visualization of internal microclimatic conditions. To measure nest climates with sensors, generally, small holes are bored in the nest envelopes; however, such holes and sensors disturb the microclimates of the nest. In this study, CFD was used to calculate the airflow and the thermal field under steadyand transient-state conditions.

**Figure 2.**
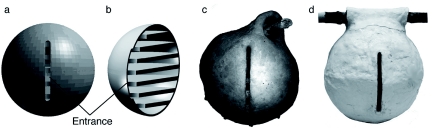
Virtual 3D-models used in this study (a, b) simulating a *Polybia spinifex* nest (c), and the model nest made of gypsum (d). High quality figures are available online.

**Figure 3.**
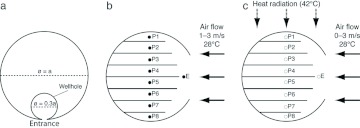
Schemes of the simulations and experimental measurements: (a) scheme of a comb with wellhole, (b) airflow conditions in Sim1 and Sim2, and (c) heating conditions in Sim3. Airspeed and temperature were measured at the center between each comb and near the entrance. Temperatures were measured at 9 points: 8 points from top (P1) to the bottom area (P8) at the centre point of combs, and I point at the nest entrance (E). High quality figures are available online.

For the numerical simulations, virtual 3Dmodels (hereafter, VtM) simulating the *P. spinifex* nest were prepared (Figures 2A, 2B) on the basis of a *P. spinifex* nest (Figure 2C; see Hozumi et al. 2009). The spherical envelope (ø = 22 cm; thickness 0.4 cm) had an entrance (2, 4, 6, 8, 10, and 12 cm in height, 0.5 cm in width) on the lateral side and contained 7 combs. Each comb was a column (1 cm in thickness) with a rounded tip to form a wellhole: a hole (30% in diameter of each comb) was bored at the entrance side (Figure 3A). The thermal properties of the nest were based on those reported by Hozumi et al. (2009): the heat conductivity (W/m·K) and the radiation rate (emissivity) of VtM were set at 0.67 and 0.80, respectively.

The numerical code PHOENICS version 2007 (CHAM Ltd., www.cham.co.uk) was used for calculations. The domain size of the current simulation was 50 cm × 50 cm × 50 cm (X, Y, Z), and the calculations were done for each grid which are defined by dividing the domain into 120 cells along the X, Y, and Z axes, respectively (1,728,000 cells in total). A κ-ε two-layer model, one of the low-Reynoldsnumber turbulence models (Launder and Spalding 1974), was used; this model is well converged unlike other turbulence models in the PHOENICS package, such as the κ-ε model, its variations, and other low-Reynolds turbulence models. For the radiation model, the IMMERSOL model of PHOENICS was employed, and the buoyancy effect was applied with the Boussinesq approximation in the moment equation. Numerical tests were also carried out to ensure that the results were independent of the number of iterations. Under-relaxation was often necessary in order to achieve convergence which was declared when the cumulative residuals for each of the conservation equations was less than 10^-3^.

### Ventilation and thermal effects of entrance lengths

In order to understand the ventilation and thermal effects on the nest due to the entrance, three kinds of simulations were carried out. First, to visualize the airflow inside the nest, air was blown through the VtM from the direction of entrance (Sim1), and the airspeed at the entrance was calculated (at the center point, 3 cm apart from the entrance, see Figure 3B). Airspeeds were varied from 1 to 3 m/s, simulating a breeze condition; although wind speed was not carried out in the field, these values are possible around the nesting sites, such as high branches in the forest and riverside. This was calculated under steadystate conditions. Second, to know the ventilation effect by the entrance length, the temporal change in airflow was calculated under transient-state conditions (Sim2). Air was blown through the VtM at 1 m/s for 10 s, and the volume where the air was displaced in the model was calculated every second. The ventilation rate (%) was estimated as follows: dividing the volume of the displaced air due to blowing by the whole nest volume. Finally, the thermal effect due to the entrance was calculated under steady-state conditions (Sim3). VtM was heated from above, and the heating temperature was set at 42° C (Figure 3c), which was the maximum value measured outdoors as reported by Hozumi et al. (2009). During the heating, air was blown through the VtM at 0–3 m/s, and the internal airflow and thermal conditions were calculated. After the simulations, the microclimates were visualized using PHOENICS version 2007.

In order to determine the reliability of the numerical results, the airspeed and temperatures in VtM were compared with model nests and with a *P. spinifex* nest, respectively. The model nests were constructed with gypsum (hereafter, GpM, Figure 2D) and the lengths of the entrance were in the 2–12cm range. A small hole was bored near the entrance of each GpM, and a hot-wire anemometer (Kanomax, Climomaster model-6543) was inserted (Figure 3B). Then, the hole was sealed with clay to eliminate gaps between the sensor and GpM. Since the thermal properties of gypsum were different from those of mud, a *P. spinifex* nest with a 12-cm entrance was subjected to temperature measurements (Figure 2C). The nest was heated from above (42° C), and the temperatures were measured at the top area, a centre point of the comb (P1), the middle area (P4), bottom area (P8), and at the entrance (Figure 2B). The experimental conditions for measuring airspeed and nest temperature were determined based on Sim1 and Sim3, respectively. The environmental temperature in both simulations and in the laboratory was 28° C, the mean ambient temperature during the day in tropic ecozones, such as Buriticupu (04° 20′ S, 46° 24′ W), Maranhafio state, Brazil, where the *P. spinifex* nest had been collected.

## Results

### Reliability of numerical models

**Figure 4.**
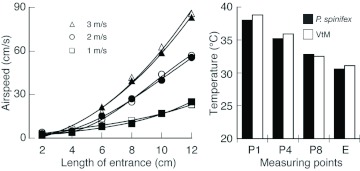
Comparison between the calculated values in VtM and measured values in GpM and a *Polybia spinifex* nest, (a) airspeed at entrance in VtM (white symbols) and GpM (black symbols); (b) temperature at P1, P4, P8, and entrance (see Figure 2) in VtM (white bars) and *P. spinifex* nest (black bars). High quality figures are available online.

Figure 4A indicates the airspeed at the entrance of VtM and GpM. The airspeeds were similar and the difference was less than 5% between each entrance length. Furthermore, the temperatures in the VtM and *P. spinifex* nests were also similar, and the difference was less than 1° C (Figure 4B). Thus, the results of the simulation based on CFD were similar to those of the experimental measurements, and it was considered that the results of simulations were representative of the microclimates in the *P. spinifex* nests.

### Ventilation effect of the entrance

**Figure 5.**
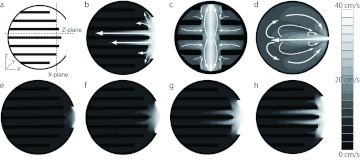
Airflow distribution in the cross section of VtM: (a) cross section viewed from the Y-plane, (b–d) visualized airflow cross-sectioned from Y-, X-, and Z-plane in the VtM with a 12-cm entrance, (e–h), flow distribution in VtM with 6, 8, 10, and 12 cm entrances, respectively. High quality figures are available online.

**Figure 6.**
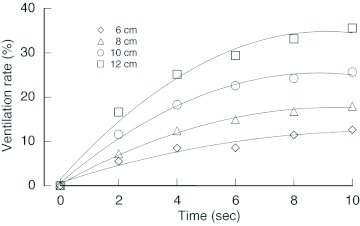
Temporal changes in the ventilation rate (%) for 10 s. High quality figures are available online.

In VtM, the airspeed decreased with entrance length (Figure 4A); whereas the airspeed was less than 5 cm/s and little airflow was observed in VtM with 2–4 cm entrances. Therefore, hereafter, the microclimates in VtM having a > 6 cm entrance were focused on. Figure 5 represents the airflow (forced convection) inside the envelope. In each VtM, the airflow branched in both vertical directions from the middle of the entrance; then, the flow further branched in the horizontal direction into the space between combs (Figure 5B, 5C). The air then returned along the envelope to the entrance (Figure 5D). The airspeed gradually dropped as the air left the entrance. Horizontal flow, however, was not observed around the top and bottom combs, which were the furthest from the entrance. When the airflow was compared among the models, both the airspeed, as well as the area where airflow was observed, were greater in the case of VtMs with longer entrances (Figures 5E, 5F, 5G, 5H). Figure 6 shows the temporal changes in the ventilation rate (%) for 10 s. The air displacement occurred in the areas where airflow was observed (see Figures 5E, 5F, 5G, 5H). The rate was always higher in VtMs with longer entrances; in the case of the 12-cm VtM, the value was 3 times higher than that of the 6-cm VtM.

### Thermal effect of the entrance

**Figure 7.**
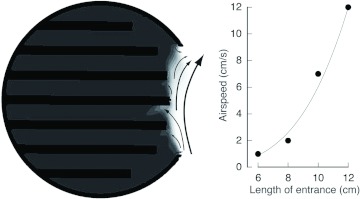
Visualization of thermal convection in VtM (a) and the airspeed at the entrance (b). High quality figures are available online.

**Figure 8.**
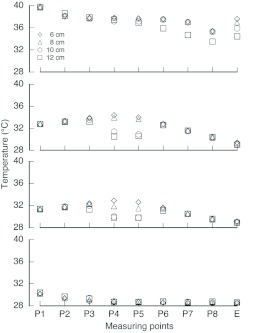
Temperature distribution in VtM: (a) calm conditions (0 m/s), (b–d) airflow conditions of 1, 2, and 3 m/s of blowing, respectively. High quality figures are available online.

Under calm conditions (0 m/s), thermal convection was observed in the VtM around the entrance (Figure 7A). The cool air entered from the lower part of the entrance, and the warm air rose though the wellhole and left from the upper part of entrance. Little airflow was observed around the mid and deeper section of the nest. The convection was stronger in the VfMs with longer entrances. On the other hand, the values were small and negligible in the case of the VtM with shorter entrances (Figure 7B). In the case of the 12cm VtM, the convective airflow dispersed the internal heat through the wellhole, and hence, the temperatures decreased in the lower combs (P6–P8) (Figure 8A). The temperature at the entrance was lower in the nests with longer entrances, whereas the temperatures at the top (P1–3) and bottom sections (P6–8) were almost similar, despite the length.

When air was blown into the VtM, the temperature at the mid combs (P4 and P5) remarkably dropped in the 10-cm and 12-cm VtMs at an airflow of 1–2 m/s by forced convection (Figures 8B, 8C). However, the temperatures at the top and bottom sections were similar among the 4 models. At an airflow of 3 m/s, all the temperatures decreased to ambient levels, i.e., the heat irradiated from above on the nest surface was removed before conducting to the interior by the external airflow. Under the airflow conditions in Sim3, the values of airspeed were similar to those in Sim1, suggesting that there was only slight ventilation due to thermal convection when air was blown into the models.

## Discussion

The characteristic entrance of the *P. spinifex* nests certainly affected the ventilation and thermal conditions of the nest. The calculations of ventilation had been done for only one case where the air was blown toward the VtM entrance. Hence, the results of airspeeds were maximum values in those boundary conditions. However, it is quite possible that the tendency that longer entrances promote ventilation will be observed, even if the wind direction varies.

A longer entrance generated more airflow when air was blown into the nest, suggesting that ventilation was automatically promoted. The entrance also partly functioned in thermoregulation by decreasing the temperature. Under calm conditions, in 12-cm VtM, the temperatures dropped from the mid to the bottom combs due to thermal convection by insolation. On the other hand, the ventilation effect due to thermal convection seemed small. When the airflow through the nest was 1–2 m/s, the temperatures at the mid combs (P4, P5) dropped due to forced convection, whereas the temperatures at the other points were similar, despite the length. This implies that in the upper (P1–3) and lower sections (P6–8) of the nest, which were the furthest from the entrance, a similar amount of heat was eliminated by thermal conduction through the envelope rather than by forced convection. At an airflow of 3 m/s, most of the heat was dispersed from the envelope surface before it was conducted into the nest. These results suggest that the ventilation can be automatically accomplished by the natural wind. The thermal convection was seen only in the calm condition. Furthermore, themoregulation by the entrance was small, and the temperature was dropped by mud envelope with a high thermal conductivity, rather than by ventilation through the long, slit-like entrance.

Generally, the ecological requirements in social wasp nests are different in different ecozones, such as the temperates, tropics and subtropics. The major factors leading to nest destruction in the tropical ecozones are attacks by vertebrate predators (Windsor 1976) and heavy rain (Richards 1978); building nests with mud may be an adaptation to these factors (O'Donnell and Jeanne 2002). In addition, in the tropic ecozone, where the ambient temperature is constantly high throughout the year, nest overheating is also an important ecological problem (Hozumi at al. 2005).

The nests of *P. spinifex* are often found in exposed situations, whereas other *Polybia* mud nesters build on high branches in the forest. It is suggested that the *P. spinifex* nests are exposed to higher insolation than other *Polybia* mud nesters. However, in terms of preventing the nest overheating, the thermal properties of the mud nest and the long entrance may be adaptations to the tropical ecozone climate. In the nests of *P. spinifex*, overheating can occur mainly due to the sun and thermogenesis by colonial activities.

When a *P. spinifex* nest is overheated, the nest is cooled by a combination of following three mechanisms: (1) thermal convection, (2) ventilation and heat removal by natural wind, and (3) thermoregulation by adult wasps. Thermal convection occurs under calm conditions, and it is possible that the temperature drop is not adequate; further ventilation alone is not enough. In this situation, active regulation, i.e., ventilative fanning and cooling activities by adults, are important to maintain suitable nest conditions. On the other hand, when the nest is blown by natural wind, ventilation is immediate, and the nest temperatures drop steeply due to convection through the entrance; however, a major portion of the temperature drop is due to emitting heat through the mud envelope with high thermal conductivities rather than a paper nest (Schmolz et al. 2000). The nests are often built at open sites; it is possible that those nests are frequently air blown by natural wind and that the nest placement is relevant to nest design, which may be related to the nest climate regulation. In *P. spinifex*, which has a large colony mass, it is possible that a stable and high nest temperature is maintained in active nests. It is possible that the microclimates in these nests have to be frequently regulated because of high CO_2_ concentration and a high temperature as a result of colonial activities. It is concluded that the long entrance in mud nests can be adaptive to maintain suitable nest climates in tropical ecozones.

In many social wasps, the shapes of the nests are strongly related to both the ecology of colonies and the regulation of internal microclimates (Matsuura 1990). The shapes of the entrances also vary with the climate, locality, and ecological conditions. Most wasps, which build envelopes around the combs, such as wasps belonging to the genus *Vespa, Vespula*, and *Polybia*, make a small hole as an entrance, in order to restrict the entry of predators and parasitoids (Spradbery 1973).

The positions and numbers of the entrances are also important for nest climate regulation; options for entrances include (1) upper, (2) bottom, or (3) two or more entrances at opposed sides of the nest. In the case of (1), the warmed air in the nest is automatically released, promoting ventilation, but the nest is filled with water when the nest has rain. In the case of (2), warmed air is stored at the top areas, and weak ventilation occurs only around the entrance. In the case (3), ventilation is increased further. For example, *Vespa basalis*, which inhabits the tropics, constructs more than 4 vertically long slits on the lateral sides of the envelope (Yamane 1992). Such entrances may promote ventilation compared with single-entrance nest of *P. spinifex*. However, in *P. spinifex* nests, multiple-entrances may decrease the colonial production because brood cells are reduced around the entrance by making convex combs (see Figure 3A).

**Figure 9.**
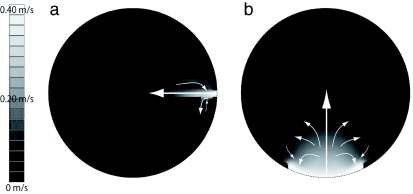
Airflow distribution in the cross section of VtM with a horizontally long entrance: (a) visualized airflow crosssectioned from Y- and (b) Z-plane in the VtM with a 12-cm entrance. High quality figures are available online.

On the ecological function of the long entrance, it is also possible that the large colony of wasps face “traffic jam” problems at the nest entrance that requires a large entrance, allowing easy access to the combs. Such an ecological function can be obvious in the nests of *Polybia scutellaris*, which also builds long entrances, but the entrance is horizontal (Hozumi et al. In press). The entrance is only connected to the outermost comb, and it is expected that the ventilation effect seems is small. In the vertical entrance of *P. spinifex* nest, however, it is possible that the entrance serves both functions, climate regulation and easy access. If the entrance is horizontal in the *P. spinifex* nest, the ventilation occurs only around the entrance (Figure 9).

This study has examined the nest microclimates and reports the ventilation and thermal functions of the nest architecture. The techniques of CFD offer new perspectives to gain a further understanding of climate regulation by nest architecture; however, more environmental parameters with regard to microclimates should be included, such as temporal changes in nest humidity, colonial heat generation, and ventilation activity by adults. Humidity is very important for passive nest thermoregulation (Klingner et al. 2005). Further studies considering all these factors are required to understand the microclimates in the *P. spinifex* nest more realistically.

## Abbreviations

CFD: computational fluid dynamics;
GpM: gypsum model;
VtM: virtual 3-D model
